# Lignin gel emulsions for environmentally benign hair conditioning

**DOI:** 10.1126/sciadv.adr8372

**Published:** 2025-02-21

**Authors:** Fengyang Wang, Sajesh Nithianandam, Ievgen Pylypchuk, Mika H. Sipponen

**Affiliations:** ^1^ Department of Materials and Environmental Chemistry, Stockholm University, SE-10691 Stockholm, Sweden.; ^2^ Wallenberg Wood Science Center, Department of Materials and Environmental Chemistry, Stockholm University, SE-10691 Stockholm, Sweden.

## Abstract

Hair care products have complex surfactant and stabilizer compositions arising from oleochemicals, raising concerns over sustainability. Here, we show a fully biobased hair conditioner based on micellar lignin gels that stabilize emulsions with triglyceride oils. We demonstrate competitive emulsion stability, rheological properties, and performance relative to an off-the-shelf commercial product. Lignin gel emulsion with a 6% weight fraction of coconut oil effectively lubricates damaged hair, confirmed by a 13% reduction in wet combing force and validated through multiscale microscopy analysis. Notably, organic solvent-free production simplifies the ingredient list and offers an environmentally benign route for lignin utilization in hair care.

## Teaser

Biobased lignin gels stabilize natural oil emulsions for sustainable hair conditioning and a simplified ingredient list.

## INTRODUCTION

A Finnish folklore suggests that if booze, tar, and sauna fail to help, then the ailment is likely fatal. Reflecting on personal experiences where hair conditioner felt unnecessary after sauna and swimming in brown-hued lake water, the lead author began to contemplate the hair conditioning role of natural polyphenols and humic substances known for their antioxidant (*1*) and surfactant (*2*) activities. Intrigued by these attributes, we hypothesized that lignin, a precursor to humic substances, could serve as a multifunctional component in hair conditioning formulations.

Hair care products contain quaternary ammonium salts surfactants, lipids, emollients, stabilizers, antioxidants, and pH adjusters (*3*). Exposure to chemical and physical agents causes degradation in the outermost fatty acid layer of hair, rendering it anionic and vulnerable to additional damage (*4*). The mechanism of action of hair conditioners is understood based on neutralization of the negative charges on the hair cuticle (*5*–*7*). Cationic surfactants form bilayers, with the second layer adsorbing through hydrophobic interactions to form a protective surface with outward-facing hydrophilic head groups (*3*). As a result, the softness, moisture, and antistatic properties are improved, enhancing texture and ease of managing hair (*8*).

A typical hair conditioner product in the retail store contains 20 to 30 ingredients, mainly derived from oleochemicals and petroleum (table S1). Driven by the expanding global hair care market (*9*) and rising consumer awareness of sustainable personal care, there has been a growing interest in natural hair care products (*8*). Hydrolyzed proteins and polysaccharides derived from plants and biotechnological processes have garnered interest (*10*). In addition, a wide range of natural oils serve as potential moisturizers and emollients, reducing moisture loss from hair (*11*). Tannins have also gained attention as surfactants in cleansing products (*12*). Many lignin grades have antistatic (*13*), antioxidant (*14*), and amphiphilic (*15*) properties, contributing to its tendency to reside at oil/water interfaces (*16*). Colloidal lignin particles stabilize oil-in-water emulsions (*17*–*19*), adsorb proteins (*20*), enzymes (*21*), and polysaccharides (*22*), increasing their ability to stabilize triglyceride oil emulsions in water (*22*). However, there is a notable gap in the literature regarding the use of lignin in hair conditioner formulations.

Here, we report a fully biobased hair conditioner based on triglyceride oil-in-water emulsions stabilized in physical lignin gels. We show that the lignin-based hair conditioner formulation can closely match a commercial product’s rheological properties and hair conditioning action, as evidenced by combing force measurements and multiscale microscopy analysis of the hair specimens. In addition, despite its intense color, our results demonstrate that this lignin-based natural hair conditioner is readily washable from hydrophilic and hydrophobic surfaces with cold water.

## RESULTS

### Lignin gel emulsion hair conditioner

**Fig. 1.**
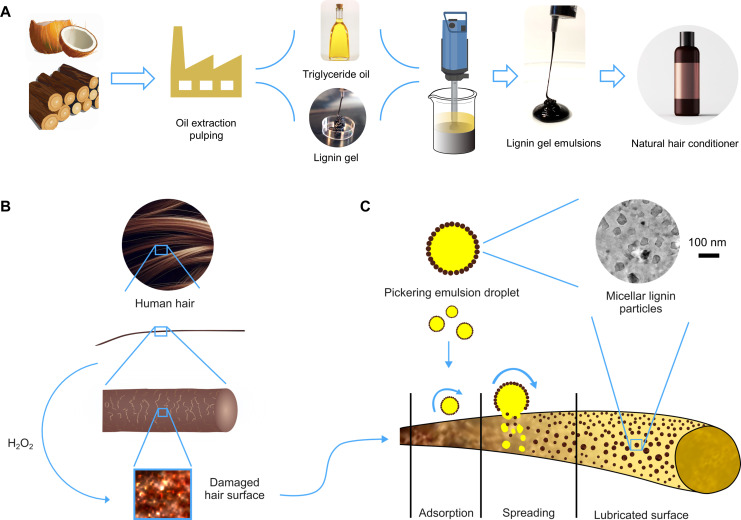
Overview of the preparation of the lignin gel emulsion hair conditioner and its working mechanism on oxidatively damaged hair. (**A**) Schematic illustration of the preparation of the lignin gel emulsions. (**B**) Damaging action of hydrogen peroxide on the human hair at the nanoscale and hair conditioning action of the lignin gel emulsion: (**C**) Amphiphilic lignin micelles present on oil droplet surfaces adsorb on the damaged hair, releasing the triglyceride oil that spreads and lubricates the hair surface. The inset cryo–transmission electron microscopy image shows the micellar lignin particles in water.

Our lignin gel emulsion hair conditioner is based on high-consistency lignin hydrogel that stabilizes Pickering emulsions with natural triglyceride oils upon mechanical homogenization (Fig. 1A). Setting it apart from chemical lignin gels, our lignin hydrogel is a physical gel of micellar origin, obtained through coprecipitation of softwood kraft lignin in the presence of sodium lignosulfonates. Sunflower oil and coconut oil were compared as the oil phase; here, we focus on the system with coconut oil. Regardless of the oil type, the emulsions were colloidally stable, viscous liquids that could flow through a syringe with applied finger pressure. To assess their hair conditioning action, we treated human hair with hydrogen peroxide, giving rise to surface-damaged hair (Fig. 1B). We hypothesized that the lignin gel emulsion could revitalize damaged hair through the combined action of micellar lignin and triglyceride oil, with the lignin micelles acting as surfactants that help spread the oil on the hair (Fig. 1C). This hypothesis is based on our experience with the interfacial stabilization of hydrophobic polymer matrices using colloidal lignin particles (*23*, *24*).

### Appearance, properties, and stability of lignin gel emulsions

**Fig. 2.**
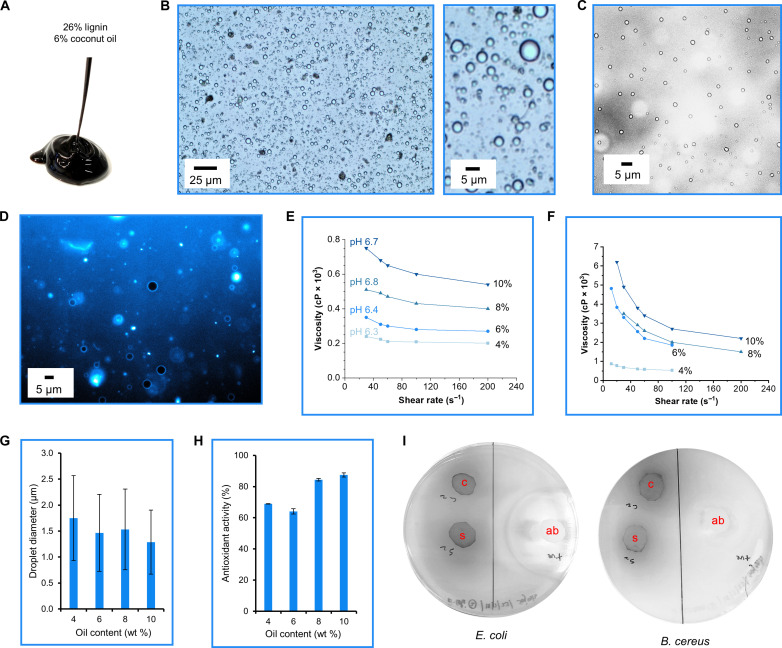
Appearance, microscopic analysis, rheology, and functional properties of the lignin gel emulsion. (**A**) Appearance of the gel emulsion with coconut oil extruded from a syringe with a nozzle head diameter of ~1 mm. (**B**) Representative optical microscopy images of the lignin gel emulsion. (**C**) A representative bright-field microscopy image. (**D**) A representative fluorescence image showing lignin autofluorescence in bright blue. The lignin content was 26 wt % in all gels characterized in this figure. Effect of coconut oil content (relative to the total weight of the emulsion) on dynamic viscosities of the emulsions (single replicates at various oil contents) at (**E**) near-neutral pH (fig. S4, A and B, for the corresponding emulsions with sunflower oil) and (**F**) pH 5.5. (**G**) Droplet diameter of the lignin gel emulsions with various oil contents based on image analysis of optical microscopy images (fig. S1). Error bars denote ±1 SD from the mean values (*n* = 100). (**H**) Antioxidant activity of the gel emulsions with various oil contents. Error bars denote ±1 SD from the mean values (*n* = 3). (**I**) Digital photographs of petri dishes from antibacterial tests showing no inhibition region around the emulsion samples (marked with c for coconut oil and s for sunflower oil) in contrast to antibiotic control (marked with ab).

The relative concentrations of lignin and oil components influenced the appearance of the Pickering emulsions. On the basis of our previous work, we anticipated the gelation point of the lignin gel to be between 25 and 27 wt % lignin content (*25*). Therefore, we used 26% lignin content as the base formulation for the hair conditioners. The appearance of the gel did not visibly change upon the addition of 6% coconut oil (weight percentage relative to the total gel weight) (Fig. 2A), but a combination of lower lignin content with a higher oil content produced gels with lighter brown appearance (fig. S1). Optical microscopic analysis revealed that the emulsion with 6% coconut oil contained a cleaner water phase in which the oil droplets were dispersed (Fig. 2, B to D, and fig. S2 for comparison of coconut oil and sunflower oil emulsions) compared to the case of the more complex commercial product that showed viscous matter in the water phase (fig. S3). The fluorescent microscopy image in Fig. 2D shows that the lignin gel stabilized a Pickering-type emulsion with lignin particles (bright blue) enriched at the water/oil interface of the oil droplets (black).

Rheological properties and stability of the emulsion are central for ensuring consumer acceptance and prolonged shelf life. Most commercial hair conditioners are adjusted to pH 5.5, the natural pH of human skin, which, in our case, also increases the viscosity of the emulsion (Fig. 2, E and F). Although the emulsions with coconut oil and sunflower oil shared, in common, this general pH dependency, the former exhibited slightly lower viscosities (Fig. 2F and fig. S4, A, B, and D), which is rationalized on the basis of the differing melting points of the two oils. Regardless of the type of triglyceride oil, the emulsions showed similar appearance and droplet size distributions, with most droplets falling to the size range of 1 to 4 μm (Fig. 2G and figs. S5 and S6). The emulsions exhibited increasing viscosities within the first 7 weeks of storage, after which it plateaued to the level of the commercial hair conditioner (fig. S4C). Rather than undergoing coalescence of the oil droplets, the change of viscosity over time originates from regelation of the system since the homogenization perturbed the original lignin gel associated with salt ions.

Commercial hair conditioners contain antioxidants that inhibit the development of rancidity in the formulation. Lignin is a natural antioxidant owing to its phenolic hydroxyl groups. Assessed on the basis of the decolorization of the 2,2′-azino-bis(3-ethylbenzothiazoline-6-sulfonic acid) radical cation solution (ABTS^·+^), all lignin gel emulsions showed considerable antioxidant activities (Fig. 2H). Increasing the sunflower oil content in the lignin gel emulsion maintained antioxidant activity at 60 to 70%, as measured by the discoloration of the ABTS radical cation solution. However, antioxidant activity increased for coconut oil from 60 to 70% to 80 to 90% when oil content rose from 4 to 6% to 8 to 10%. In contrast to the evident antioxidant activity, the lignin gel emulsion did not show antibacterial activity against *Escherichia coli* or *Bacillus cereus* (Fig. 2I and fig. S7). Including a lignin-derived antimicrobial agent, such as those obtained from lignin pyrolysis oil (*26*), would therefore be beneficial.

### Hair conditioning with lignin gel emulsion

**Fig. 3.**
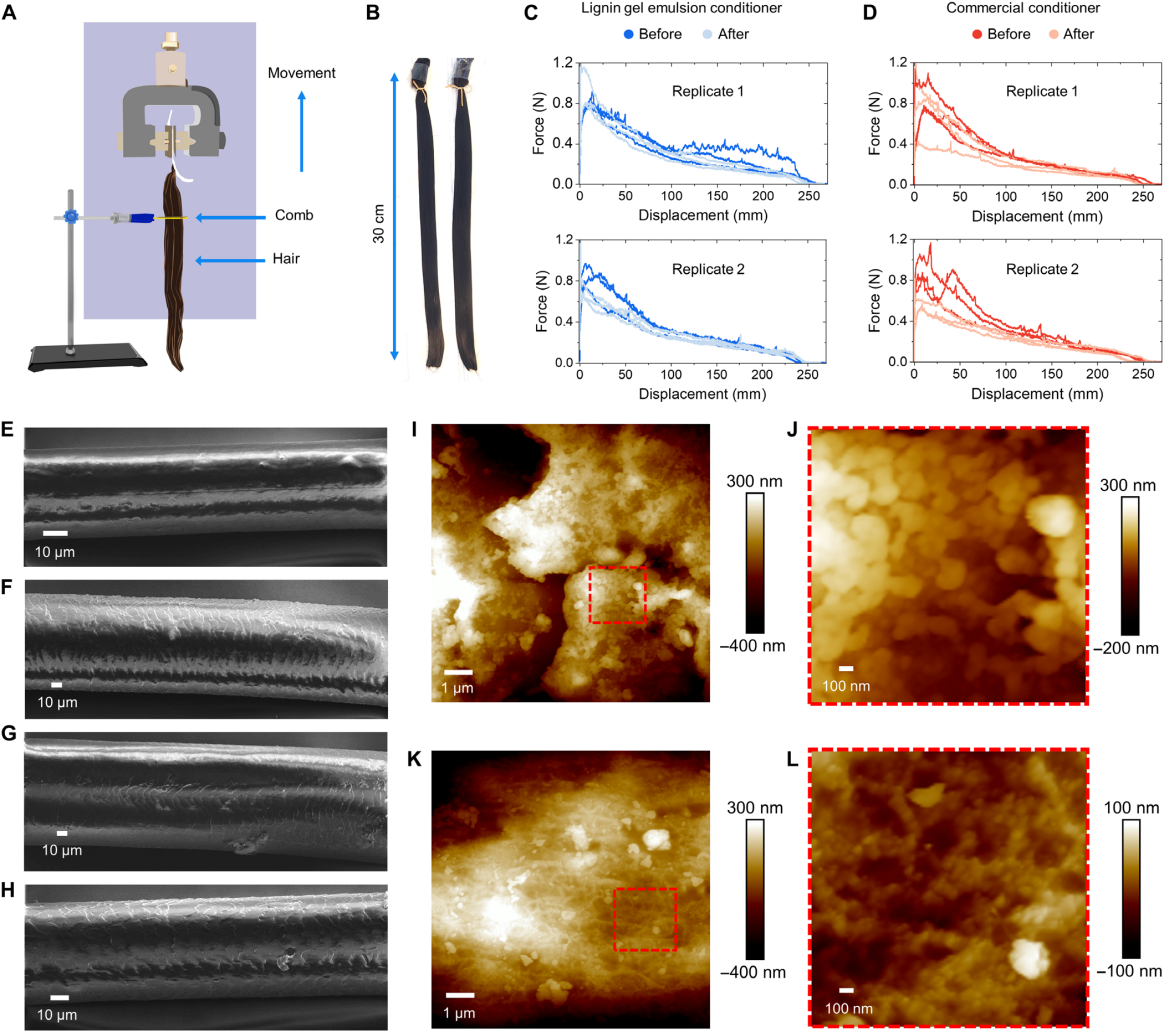
Hair conditioning with lignin gel emulsion conditioner. (**A**) Setup for measurement of wet combing forces. (**B**) Appearance of the hair tresses used in the experiments. Combing forces of two replicate tests before and after conditioning of peroxide-oxidized hair with (**C**) 6% coconut oil in lignin gel emulsion and (**D**) commercial hair conditioner. Scanning electron microscopy (SEM) images of hair used in combing force measurements: (**E**) unbleached hair and (**F**) hair damaged with 6% aqueous hydrogen peroxide. Damaged hair treated with (**G**) the lignin gel emulsion conditioner and (**H**) commercial conditioner. Atomic force microscopy images of (**I** and **J**) damaged hair conditioned with lignin gel emulsion and (**K** and **L**) damaged hair conditioned with the commercial hair conditioner.

The lignin gel emulsion with 6% coconut oil was selected for actual hair conditioning tests owing to its rheological properties resembling the commercial formulation and slightly better colloidal stability compared to the emulsion with similar content of sunflower oil (fig. S1, B and C). The tests were carried out by treating peroxide-oxidized hair with either a commercial conditioner or the lignin/coconut oil conditioner and measuring the wet combing force (Fig. 3A) of the hair samples (Fig. 3B). The lignin gel emulsion showed comparable performance to the commercial conditioner, markedly reducing the combing force (Fig. 3, C and D). Averaging over the two replicate experiments produced 13 ± 1% reduction in the wet combing force by the lignin gel emulsion compared to 20 ± 9% with the commercial formulation. There was no statistically significant difference between the performances of the two hair conditioner formulations (*t* test, *P* > 0.05). On the basis of these results, we anticipate that the lignin gel emulsion likely formed a coating layer comprising both amphiphilic lignin and coconut oil on the hair. Such an outcome would be explained by the restoration of the lubricating fatty acid layer in undamaged hair (*27*).

To understand the postulated lubricating effect of the hair conditioner, we used scanning electron microscopy (SEM) to investigate changes on the hair surfaces. The untreated hair filament showed smooth surface morphology, with low extent of charging in contrast to the peroxide-bleached hair (Fig. 3, E and F, and fig. S8). Conditioning the peroxide-bleached hair with the lignin gel revitalized the surface morphology to a comparable extent to that of the commercial conditioner (Fig. 3, G and H). A closer investigation of hair treated with the lignin gel emulsion and a commercial conditioner revealed comparable smoothing effects on the hair surface. However, the lignin gel emulsion does not deposit micrometer-scale particles on the hair surface, supporting our initial hypothesis that the Pickering emulsion droplets release their oil contents and form a lubricating composite coating embedding lignin on the hair surface (compare Fig. 3, I and J and K and L).

Pickering emulsions offer surfactant-free emulsions that, when applied to human skin, are perceived as less greasy and nonsticky than conventional surfactant-stabilized emulsions, especially with higher oil loading (*28*, *29*). Different lignin grades are gaining interest in sunscreen formulations (*30*–*34*), owing to their surface activity and emulsion stabilization properties (*35*–*39*). In a broader context, the formation of cation complexes renders lignosulfonates into effective stabilizers of crude oil in water emulsions (*38*). Another study showed that adding potassium chloride into a solution with sulfethylated kraft lignin reduced surface tension at xylene, cyclohexane, and decane interfaces (*39*). Therefore, the salts in the lignin gel emulsions likely contributed to enhanced adsorption onto hair in the present study.

**Fig. 4.**
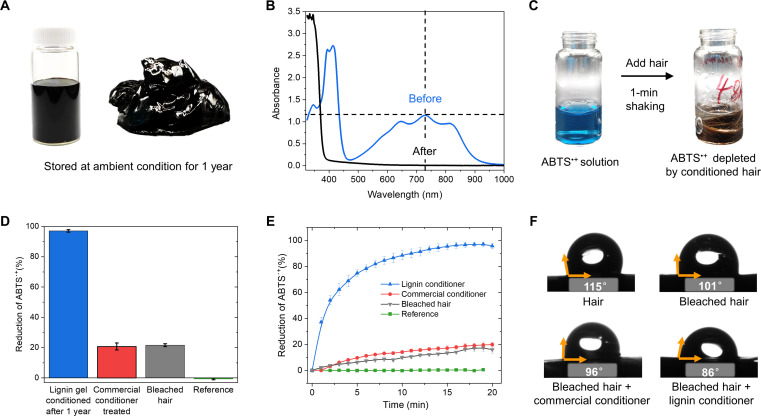
Storage stability, antioxidant activity, and the surface modification of lignin gel emulsion with 6% coconut oil. (**A**) Photographs of the lignin/coconut oil emulsion conditioner before and after 1 year storage. (**B**) Ultraviolet-visible (UV-vis) absorption spectrum of the ABTS^·+^ solution (3 ml) before and after 1-min mixing with lignin/coconut oil emulsion conditioner–treated hair (300 mg). (**C**) Antioxidant effect of lignin/coconut oil emulsion conditioner on hair, shown by the decoloration of the ABTS^·+^ solution. (**D**) Reduction in the absorbance of ABTS^·+^ solution after 1-min mixing with nontreated bleached hair (300 mg) and bleached hair (300 mg) treated with lignin/coconut oil emulsion conditioner, commercial conditioner, and ABTS^·+^ solution alone as reference. Results show mean values ± SD of three independent experimental replicates. (**E**) Kinetic study of the antioxidant activity in treated hair (15 mg), shown by reduction in the absorbance of ABTS^·+^ solution (3 ml) for 20 min. Results show mean values ± SD of three independent experimental replicates. (**F**) Immediate water contact angles of sessile drops on untreated and treated hair samples. Note that in (D), the hydrogen peroxide–treated hair was treated with freshly made lignin gel emulsion 1 year before the antioxidant activity test, while in (B), (C), (E), and (F), the hydrogen peroxide–treated hair was conditioned with 1-year stored lignin gel emulsion, dried at room temperature, and kept under ambient conditions for 48 hours before testing for antioxidant activity.

As shown in the photos of the lignin conditioner (comprising emulsion of 6% coconut oil in lignin gel) after ~1 year of storage under ambient conditions, no noticeable phase separation or creaming was observed (Fig. 4A). This good long-term stability is likely attributed to the high viscosity of the emulsion and the electrostatic double-layer force introduced by the negatively charged lignin nanoparticles. According to Stokes’ law, the drag force opposing gravitational force is proportional to viscosity, supporting colloidal stability by counteracting sedimentation. In addition, the electrostatic double-layer force moderates particle-particle aggregation, further enhancing the lignin conditioner’s stability over time (*25*). These two factors are believed to be the key parameters in strengthening the colloidal stability of coconut oil in lignin gel emulsion. To further assess how the lignin conditioner would affect hair care after long-term storage, we performed the ABTS^·+^ assay on bleached hair treated with the lignin gel, which was stored under ambient conditions for 1 year. As shown in Fig. 4 (B and C) and movie S1, the treated hair depleted ABTS radical cations after 1 min of contact, reducing the absorbance at 734 nm from 1.1 to 0. This rapid reduction suggests that the antioxidant properties of the lignin conditioner remain active even after extended storage. In addition, it is also important to understand how long the antioxidant property will remain effective on hair after applying the conditioner. Therefore, we performed the ABTS^·+^ assay on bleached hair that was previously conditioned with the lignin conditioner and stored for around 1 year. For comparison, the antioxidant assay was also performed on bleached hair treated with a commercial conditioner (movie S2), as well as untreated bleached hair and reference sample. After 1 year of storage, lignin conditioner–treated hair still exhibits strong antioxidant capacity, showing a nearly 100% reduction of ABTS^·+^ after 1-min contact with the liquid (Fig. 4D), performing equally compared to freshly conditioned hair. In contrast, the reduction of ABTS^·+^ for bleached hair and commercial conditioner–treated bleached hair was markedly lower at around 21%.

To better understand the kinetics of this antioxidant activity, we repeated the ABTS^·+^ assay with smaller quantities of hair (15 mg). The kinetic analysis revealed that the ABTS radical cations were reduced by ~80% in the first 5 min and then slowly reached the plateau after 20 min, whereas the reference ABTS^·+^ solution showed negligible change in this time frame (Fig. 4E). The bleached hair treated with a commercial conditioner displayed only slightly enhanced antioxidant capacity in the kinetic study. Apart from the antioxidant capacity introduced by the particles adsorbed onto the hair, the surface hydrophobicity varied depending on the different treatments.

Oxidation alters the surface chemistry of hair, a process that leads to the need for hair care. As shown in Fig. 4F, the original hair has a contact angle of about 115°, which is reduced to about 101° after bleaching with alkaline hydrogen peroxide solution due to oxidative surface damage. The contact angle decreased to 96° and 86° for bleached hair treated with commercial and lignin conditioner, respectively (see table S2 for all water contact angles data). This increased hydrophilicity is likely due to the adsorption of cationic surfactants from the commercial conditioner. In contrast, the lower contact angle resulting from the lignin conditioner suggests the adsorption of amphiphilic lignin particles and coconut oil on the hair surface. Combining these findings from the ABTS^·+^ assay, kinetic studies, and surface hydrophobicity measurements, the results indicate that lignin particles and oil droplets from the conditioner adhere effectively to the hair surface, contributing to sustained antioxidant activity and enhanced moisturization after treatment.

**Fig. 5.**
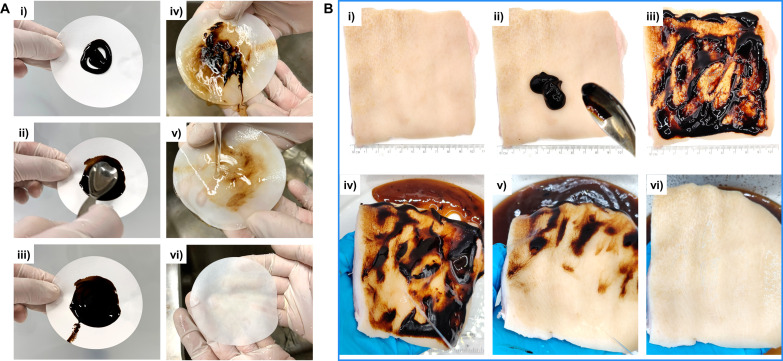
Washability of the 6% coconut oil in lignin gel Pickering emulsion. (**A**) Cellulose filter paper and (**B**) porcine skin. The emulsion was gently spread on the substrate with a spoon, followed by 1-min contact and washing within 1 min under DI water flow without rubbing.

A distinct feature of lignin-based hair conditioners is their black color, raising questions about washability and potential staining, especially on cotton towels. We tested the washability of the lignin gel emulsion from cellulose filter paper (Fig. 5A). The emulsion was spread on filter paper, left for 1 min, and then gently washed with deionized (DI) water, largely cleaning the filter paper. Literature on the adsorption and desorption of lignosulfonates on cellulosic surfaces is lacking. The most relevant investigations have studied the adsorption of kraft lignin on cellulose pulp fibers (*40*) and molecular dynamics studies of lignin dimer adsorption on cellulose model surfaces (*41*). Although the emulsion formulation is not identical to these papers, our experimental results agree with Maximova and co-workers (*40*), who reported extensive desorption of kraft lignin from cellulose pulp fibers by water washing.

The washability of the lignin-based hair conditioner on the skin is equally important as that of cotton towels. To validate this, we performed the washability test in the same manner as mentioned above using porcine skin, which is similar to human skin from histological and physiological views. As shown in Fig. 5B, there is no visually noticeable stain after the test, demonstrating that the lignin-based hair conditioner can be easily washed from porcine skin with water. Here, it is also important to emphasize that safety is central in cosmetic applications due to skin contact. In the present work, the main components of lignin conditioner, kraft lignin, and lignosulfonates are not classified as hazardous (*42*, *43*). Lignosulfonate is used in commercial products such as eyeliner, mascara, hair coloring, and bleaching (*44*). The antioxidant and dispersing properties of lignosulfonates also highlight their potential as a valuable ingredient in other personal care formulations.

## DISCUSSION

In the present work, we used micellar lignin hydrogels to stabilize hair conditioner emulsions, providing lubrication and natural antioxidant activity for hair care. The lignin gel emulsion that we developed in this study offers an environmentally benign alternative to fossil-based synthetic cationic surfactants that are common ingredients in commercial hair conditioner formulations. The lignin gel emulsion contains only water, sodium sulfate, softwood kraft lignin, sodium lignosulfonate, and triglyceride oil. Our results demonstrate that the lignin gel emulsion exhibits excellent stability after a sustained storage time of about 1 year, which ensures extended shelf life for potential commercial use. The production could also be easily scaled up since no covalent chemical modifications were required during the process. Currently, a kilogram scale in output can be easily achieved on a laboratory scale. By combining the amphiphilic properties and lubricating action of the emulsion with lignin’s well-known ultraviolet (UV)–absorbing functionality, the formulation shows promising potential for hair protection. As the demand for eco-friendly and functional ingredients rises, the potential for lignin-based formulations in the hair care market is promising, giving a unique opportunity for innovation and market growth.

Consumers are becoming self-aware of the benefits of natural cosmetics (*45*). Concerns surrounding personal care products and their ingredients include petrochemical origins, animal exploitation, deforestation, reliance on heavy metal catalysts, competition with food production, environmental pollution, nonbiodegradable or bioaccumulative materials, and harmful solvent residues (*46*). One of the critical questions relates to the selection of sustainable raw materials. On the basis of our results, it is lucrative to harness lignins as multifunctional polyphenols of natural origin. There is a growing research front on the use of lignins as SPF boosters in sunscreen formulations (*33*). Lignin-based ingredients for personal care products, including shampoo formulations, are already available (*47*). However, lignin-based hair conditioners are still awaiting commercial launch.

One notable aspect of our lignin-based conditioners is their intense coloration, which raises concerns about potential staining or discoloration. However, our washability tests on cellulose filter paper and porcine skin indicate that the lignin conditioner can be easily rinsed off without leaving residues. This suggests that the risk of staining is relatively minor in practical applications. Nevertheless, further studies could optimize the formulation to ensure compatibility with various hair types and colors. Moreover, while promising for sustainability, further research is needed to elucidate sensory attributes and safety aspects, including potential skin and eye sensitization. We anticipate that our pioneering work will inspire further research to address these challenges, expanding opportunities for lignin utilization.

Developing a fully biobased hair conditioner using lignin gel emulsions offers opportunities to create sustainable personal care products. Our research shows that micellar lignin particles can effectively stabilize oil-in-water emulsions, providing conditioning performance comparable to commercial products. In addition, lignin’s antioxidant properties and environmental safety make it an appealing, sustainable alternative in hair care formulations. Furthermore, the lignin gel emulsion formulation’s natural composition, gentle action, and lignin’s antioxidant and moisturizing properties suggest its potential for sustainable pet grooming, supporting healthy fur and skin. Targeting these market segments that include high-value products could offer compelling benefits for the biorefinery industry, potentially adding extra value to the lignin and fatty acids fractions. We foresee that these potential benefits could arouse great interest from the traditional forestry and emerging agricultural biorefinery industry. Overall, the present work highlights the expanding potential of lignins in personal care applications, contributing to the growing body of research in this field.

## MATERIALS AND METHODS

### Experimental design

The main objective of the study was to test the hypothesis that a fully biobased lignin hydrogel could serve as a base for natural hair conditioner formulations. The study design included experimental testing of the emulsification of various concentrations of sunflower oil and coconut oil in the lignin gel emulsion. In addition, the mass ratio of the triglyceride oil to the total lignin content was examined. Emulsions with visually stable characteristics were further analyzed through dynamic viscosity measurements to identify a formulation with suitable rheological properties. The coconut oil lignin gel emulsion was selected for more detailed characterization and application tests to evaluate its hair conditioning performance.

### Materials

Sodium lignosulfonate (DS10, Domsjö, Sweden), softwood kraft lignin (BioPiva 100, UPM, Finland), basic human hair extension (Fairwithhair Josiander AB, Sweden), coconut oil (pure, refined, Thermo Fisher Scientific), sunflower oil (pure, refined, Mini Ellada AB, Sweden), commercial conditioner (Elvital Color Vive, L’Oréal Paris), and a black polypropylene plastic comb of 11 cm (with a fine tooth length of 5.5 cm and a gapping of 1 mm and a wide tooth length of 5.5 cm and a gapping of 3 mm) were purchased from a local supermarket (Stockholm, Sweden). Food-grade porcine skin was purchased from a local shop in Stockholm. All other chemicals and materials were purchased from VWR (Sweden) and used as received.

### Lignin gel preparation

The colloidal lignin dispersion was prepared according to the literature (*25*). A mixture of lignosulfonate (2.1 kg; dry substance) and softwood kraft lignin (568 g; dry substance) in the ratio of 5:1 was dissolved in 2.3 liters of 2 M aqueous sodium hydroxide solution and stirred overnight. To this solution, 200 ml of DI water was added to facilitate the complete dissolution of the mixture at pH ~ 13.0. Thereafter, 900 ml of 2 M sulfuric acid was added to obtain a lignin gel at pH 5.5. This resulted in a highly viscous gel dispersion at a total lignin concentration of 37 wt %.

### Oil-in-water emulsion preparation

Emulsions with a fixed lignin concentration of 26 wt % and oil contents of 4, 6, 8, and 10% (v/v) were prepared each with a total volume of 150 ml. Initially, lignin gel was mixed with water phase, and then the oil was introduced into it. These mixtures were then homogenized for 5 min at 15,000 rpm using an IKA-T25 ULTRA-TURRAX (IKA, Germany) homogenizer. All the prepared emulsions were stored at room temperature for further analysis.

### Hair conditioning

Hair tresses were wetted with warm water for about 30 s, and excessive water was removed. Dry hair (8 g) was wetted and treated with 1.5 ml of either lignin-based coconut oil conditioner (6% oil content) or commercial conditioner. After 1-min conditioning, the tresses were washed with warm water to remove the excess conditioner, and the conditioned hair was evaluated for wet combing analysis.

### Characterization methods

#### Optical microscopy

The shape and size distribution of emulsion droplets were analyzed using a Nikon Eclipse Ti inverted optical microscope (Nikon, Japan) at ×40 magnification, equipped with a DS-Fi3 camera (Nikon, Japan). A 10-μl droplet of the diluted emulsion was placed on a microscope slide, covered with a cover slip, and visualized. Images were analyzed with ImageJ software, measuring the diameters of 100 droplets to report the mean droplet size.

#### Fluorescence microscopy

The emulsions were visualized using an inverted Axio observer Z1/7 microscope (Carl Zeiss, Germany) and a Hamamatsu camera (Hamamatsu, Japan) at ×100 magnification with oil immersion technique and with excitation at 488 nm and emission at 509 nm. A droplet of the emulsion diluted in DI water (5 μl) was deposited on the microscope slide and then covered with a cover slip before visualizing in the microscope.

#### Scanning electron microscopy

The JSF-7000F instrument (JEOL, Japan) was used for SEM. A small segment of hair sample was mounted on a sample holder grid and secured with copper tape. Samples of unbleached hair and bleached hair with and without conditioner treatment were examined, and images were captured using an accelerating voltage of 5.0 kV.

#### Atomic force microscopy

The Multimode-8 instrument (Bruker, USA) was used to acquire atomic force microscopy images, operating in PeakForce Tapping mode with ScanAsyst algorithm self-optimization. ScanAsyst-air probe was used in all measurements. All the images were processed using Nanoscope Analysis 2.0 software.

#### Dynamic viscosity

Dynamic viscosities of the emulsions were measured using a rotational viscometer, Viscotech Myr VR 3000 with R2-R5 spindles (Viscotech, Spain). Dynamic viscosities of the emulsions (150 ml each) were determined and compared against different oil content and pH.

#### Antioxidant assay

The antioxidant capacity of the emulsions was assessed using the ABTS assay. ABTS radical cation was prepared by combining 7 mM ABTS and 2.45 mM potassium persulfate and then stored in the dark for 16 to 24 hours at room temperature. Absorbance measurements were performed by using a UV–visible (vis) spectrophotometer (Genesys 150 UV-vis spectrophotometer, Thermo Fisher Scientific, USA). The solution was diluted with DI water to an absorbance of 0.85 at 734 nm at 25°C. For analysis, 2 ml of the diluted ABTS radical cation solution was mixed with 20 μl of the sample and reacted in the dark for 2 min. Absorbance at 734 nm was recorded in triplicate. The inhibition percentage was calculated using the formula: Inhibition % = (*A*_c_ − *A*_s_) / *A*_c_ × 100, where *A*_c_ is the absorbance of the ABTS and *A*_s_ is the absorbance of the sample.

For the antioxidant test of hair samples, freshly prepared ABTS solution was diluted with DI water until an absorbance of around 1 at 734 nm at 25°C; 3 ml of diluted ABTS solution was measured before and after immersing 0.3-g hair sample in the solution for 1 min of shaking; for the kinetic study, 15-mg hair and 3 ml of diluted ABTS solution were used; and the absorbance spectrum is recorded at 1-min intervals. The results are reported as mean value ± 1 SD based on three independent experimental replicates.

#### Antibacterial activity

To test the antibacterial susceptibility of the stabilized emulsion, we used the disc diffusion method with *E. coli* (CCUG 10979) and *B. cereus* (CCUG 7414). Both bacteria were cultured on LB medium and incubated at 37°C for 24 hours. The bacterial concentration was adjusted to the 0.5 McFarland standard. Then, 500 μl of the bacterial culture was spread on Muller-Hinton agar plates. Emulsions with 26% lignin and 6% sunflower oil or coconut oil were diluted 10-fold. Sterile cellulose fiber discs were dipped in the diluted emulsion and placed on the inoculated plates, with a sterile disc dipped in ampicillin as a positive control. Tests were performed in triplicate, and plates were incubated at 37°C for 24 hours to observe zones of inhibition.

#### Hair-damaging treatments

A nondyed and nonbleached human hair extension (dark brown color) with 50 cm in height and 100 g in weight was used in hair treatments. Four samples of hair tresses were prepared, each with a width of 2.5 cm, a length of 30 cm, and a weight of 8 g. The hair tresses were prewashed with DI water and then subjected to bleaching process to represent damaged hair. The hair tresses were immersed in aqueous 6% hydrogen peroxide at pH 10.2 and left for 1 hour at 40°C. After this, the tresses were washed repeatedly with copious amounts of DI water and allowed to dry overnight at room temperature.

#### Combing force measurements

Wet combing analysis was performed following the published protocol with minor modifications (*48*). The wet combing experiments were performed using an Instron 5960 series tensile tester (Instron, USA) equipped with a Bluehill software. The testing setup involved pulling the hair through the fine tooths of a comb as seen in Fig. 3A, with a scanning speed of 200 mm/min, recording the combing force. We calculated the combing force as the average force from valid data points where the hair maintained contact with the comb. Each treatment (lignin gel emulsion or commercial conditioner) included three measurements per replicate, with two replicates in total. First, we averaged the measurements within each replicate and then calculated the overall average across replicates to determine the percentage reduction in combing force. The reduction in combing force after conditioner was calculated using the given formula: Combing force reduction % = [average force (N) before conditioner − average force (N) after conditioner] / [average force (N) before conditioner] × 100%.

#### Water contact angle

Water contact angle was measured with Drop Shape Analyzer – DSA25E (KRÜSS Instruments, Germany), and 2-μl water drop was used, with three replicates. All the images were processed with the ADVANCE software.

#### Water washability

A lignin gel emulsion containing 6 wt % coconut oil (~1 g) was applied onto cellulose filter paper (Munksjö) using a spatula and left in contact for ~1 min. Subsequently, the filter paper with the emulsion was subjected to a moderate flow of DI water for 1 min without any rubbing to assess washability. Digital photographs were taken during the process to visually document the washing procedure. Skin washability was performed in a similar way mentioned above, except that 3 g of lignin gel emulsion containing 6 wt % coconut oil was applied onto porcine skin (10 cm by 10 cm).

### Statistical analysis

All data reported in this study are mean values, and, where error bars are presented, they denote ±1 SD relative to the mean. The number of experimental replicates along with the values for *N*, *P*, and the specific statistical test performed for each experiment are included in the appropriate figure legend in the main text.
