# Supplementary material for: Lignin gel emulsions for environmentally benign hair conditioning
Source: Sci Adv. 2025 Feb 21;11(8):eadr8372. doi: 10.1126/sciadv.adr8372 (PMC11844739; doi:10.1126/sciadv.adr8372)
Supplement: Supplementary file 1 — Figs. S1 to S8 Tables S1 and S2 Legends for movies S1 and S2 [file sciadv.adr8372_sm.pdf]

Science Advances
AAAS

Supplementary Materials for

Lignin gel emulsions for environmentally benign hair conditioning

Fengyang Wang *et al.*

Corresponding author: Ievgen Pylypchuk, ievgen.pylypchuk@mmk.su.se;
Mika H. Sipponen, mika.sipponen@mmk.su.se

Sci. Adv. **11**, eadr8372 (2025)
DOI: 10.1126/sciadv.adr8372

The PDF file includes:

Figs. S1 to S8
Tables S1 and S2
Legends for movies S1 and S2

Other Supplementary Material for this manuscript includes the following:

Movies S1 and S2

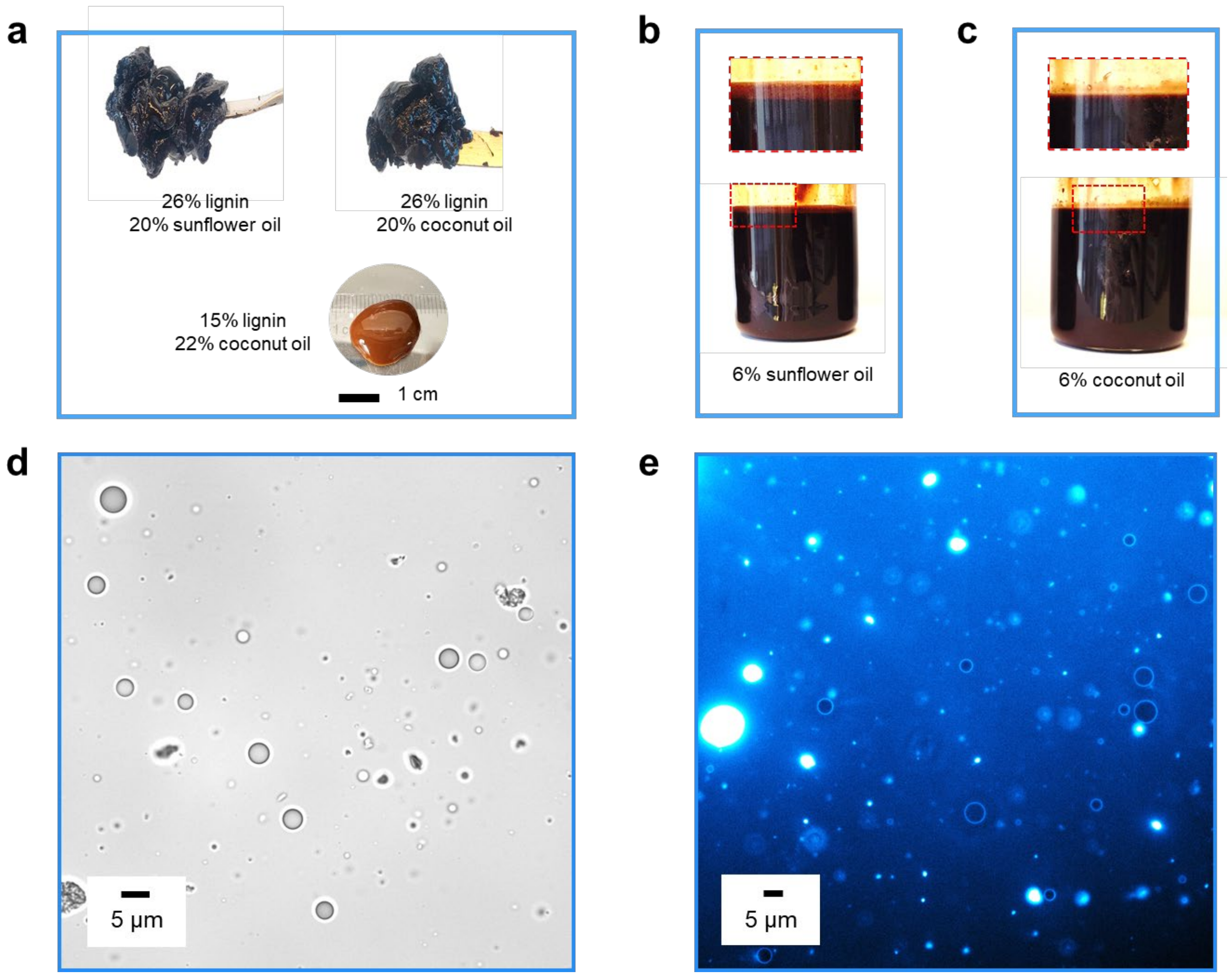

Fig. S1. Appearance of lignin gel emulsions. Digital photographs of a, emulsions with different lignin content and oil types, b-c, emulsions with 26% lignin content and 6% either sunflower oil or coconut oil after 100 days storage at room temperature. Fluorescence microscopy images of d-e, emulsion with 26% lignin content and 6% sunflower oil as the oil phase, showing autofluorescence of lignin at the Pickering droplet shells.

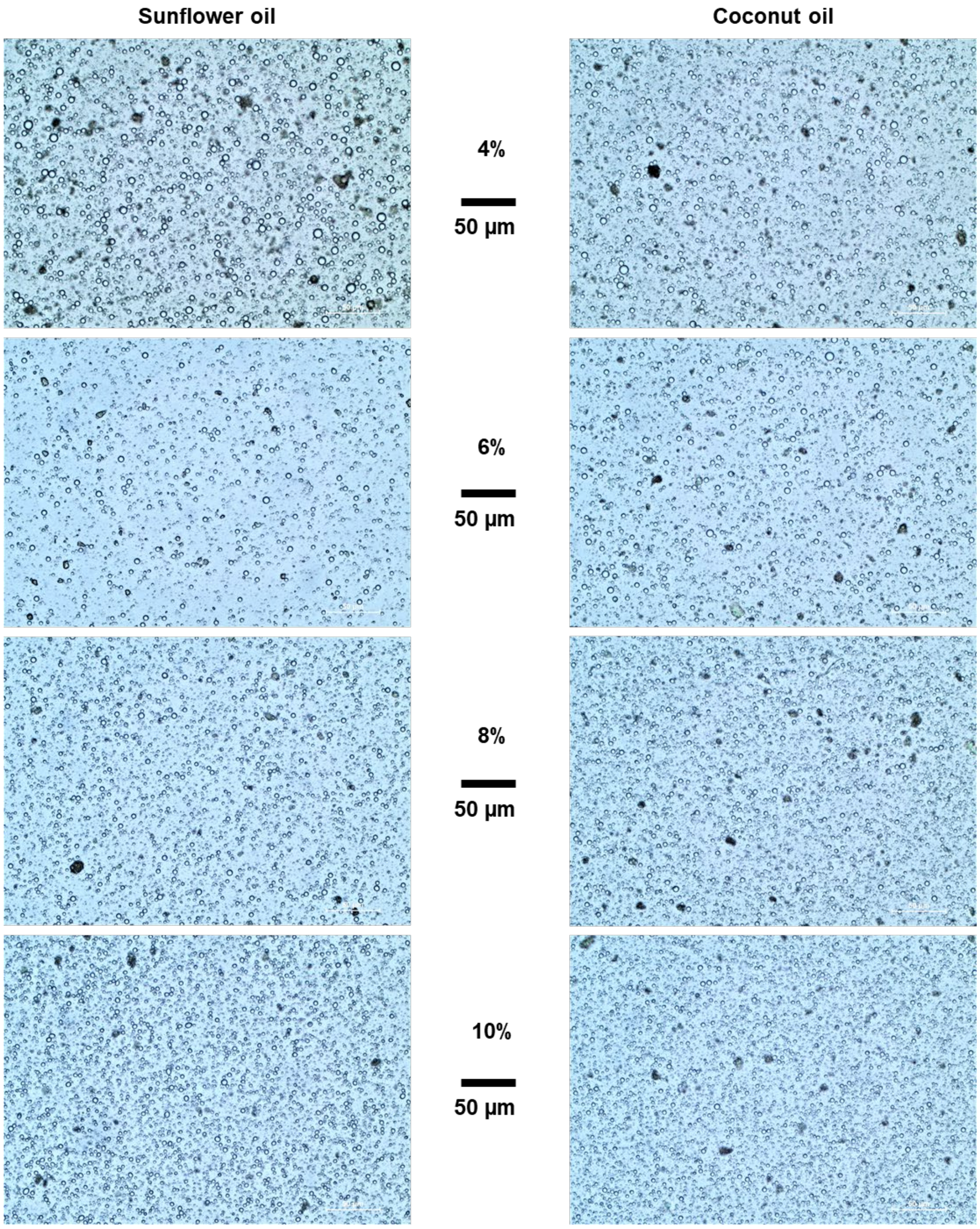

Fig. S2. Optical light microscopy images of lignin gel emulsions. All emulsions contained 26% (w/w) lignin while the oil content was varied from 4% to 10% (w/w).

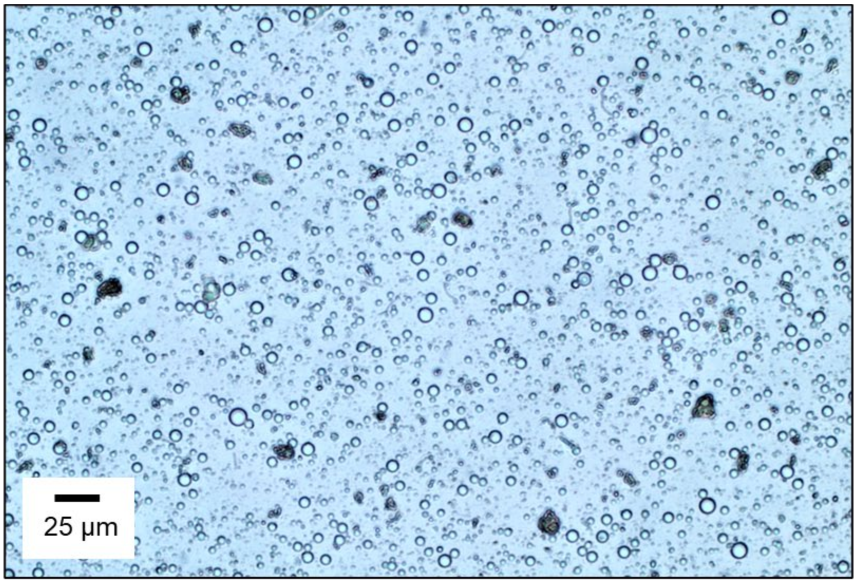

Lignin gel emulsion with 6% coconut oil

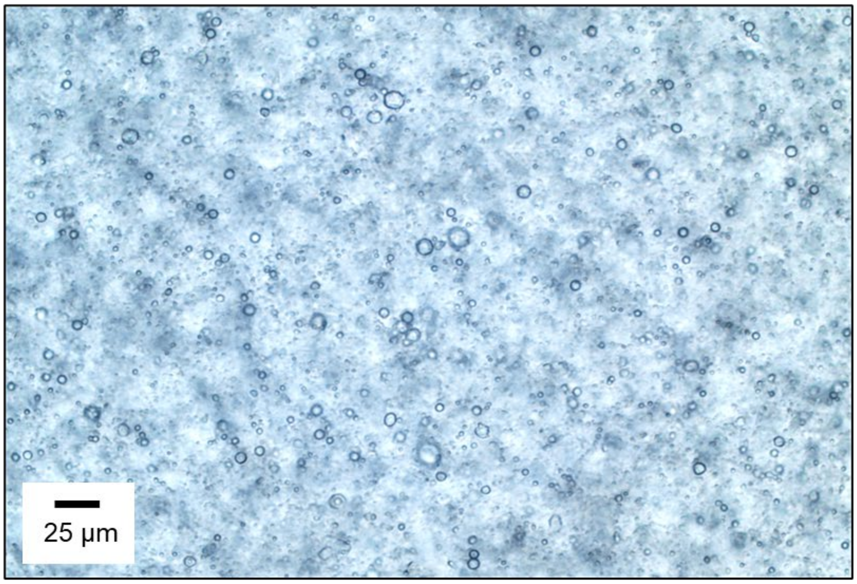

Commercial conditioner

Fig. S3. Optical light microscopy images of lignin gel emulsion and commercial hair conditioner. The lignin conditioner 26% lignin content and 6% coconut oil content (expressed as w/w).

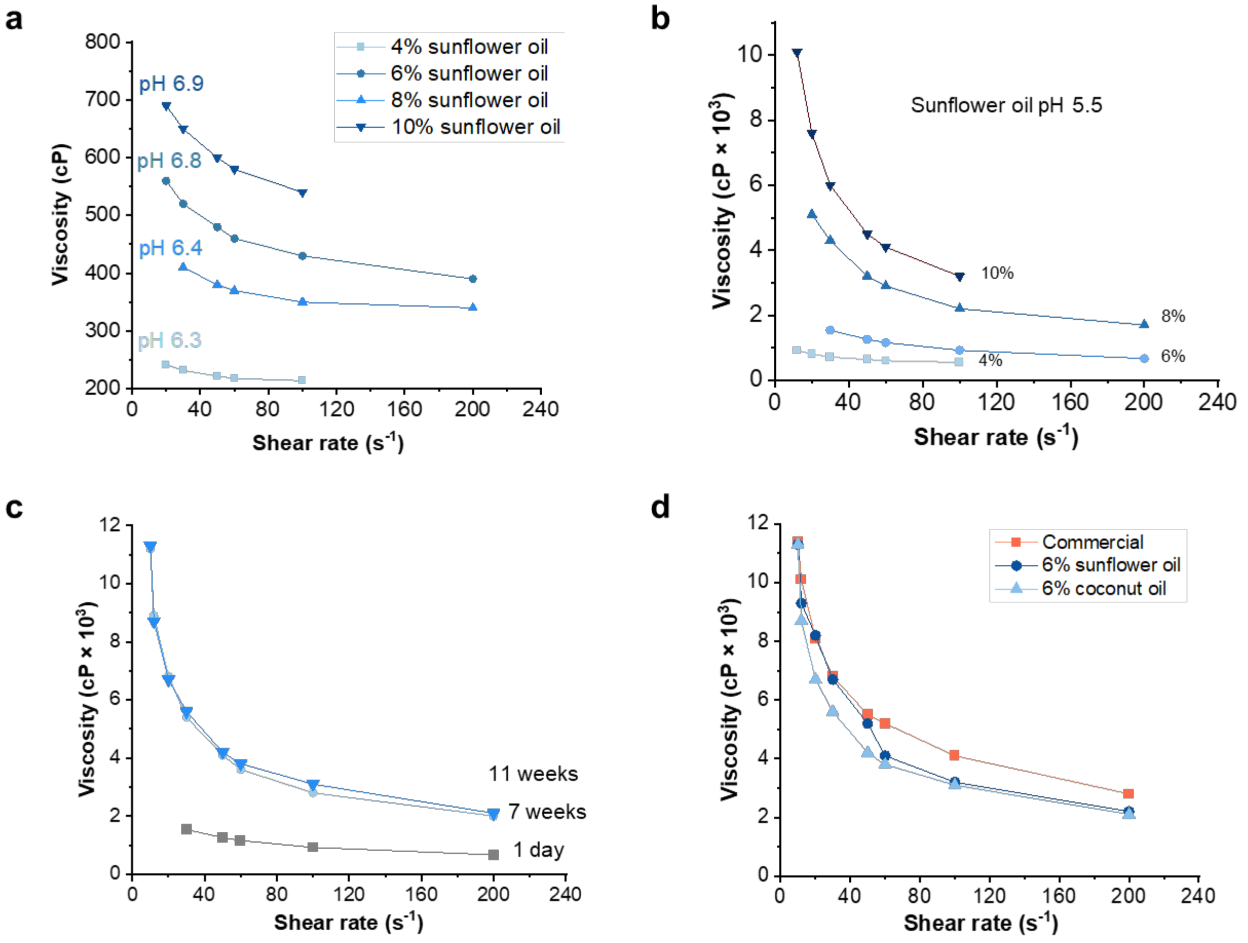

Fig. S4. Dynamic viscosities of different lignin gel emulsions. Effect of sunflower oil content (w/w) on dynamic viscosity of the emulsions at **a**, near-neutral pH; **b**, pH 5.5. **c**, Effect of storage time at room temperature on the dynamic viscosity of lignin gel emulsion 6% (w/w) coconut oil. **d**, Comparison of lignin gel emulsions after 11 weeks storage to that of commercial hair conditioner. All lignin gel emulsions included in this figure had a lignin content of 26% (w/w).

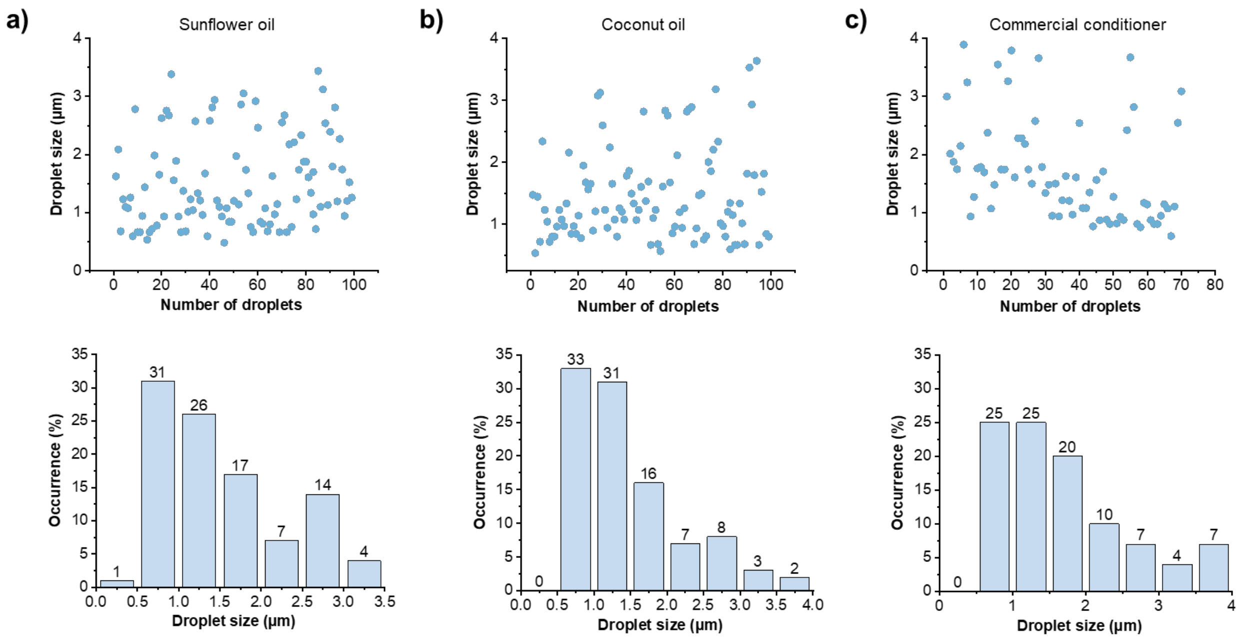

Fig. S5. The droplet size distributions of emulsion preparations. The droplet diameters were obtained through image analysis of 100 droplets from optical microscopy images. Lignin gel emulsions with oil content of 6% (a) sunflower oil, (b) coconut oil, compared to (c) The commercial hair conditioner.

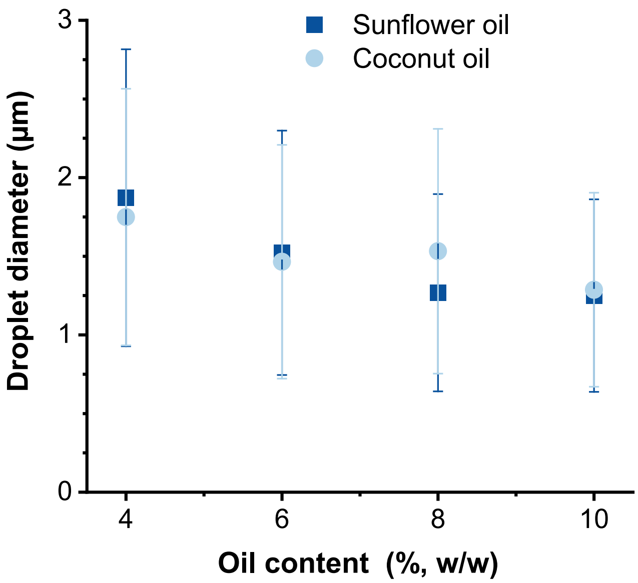

Fig. S6. The droplet size of lignin gel emulsions with varied oil content. The gel emulsions with 26% lignin (w/w) were prepared with different weight percentage of sunflower oil or coconut oil. The droplet diameters were obtained through image analysis as above in Fig. S2.

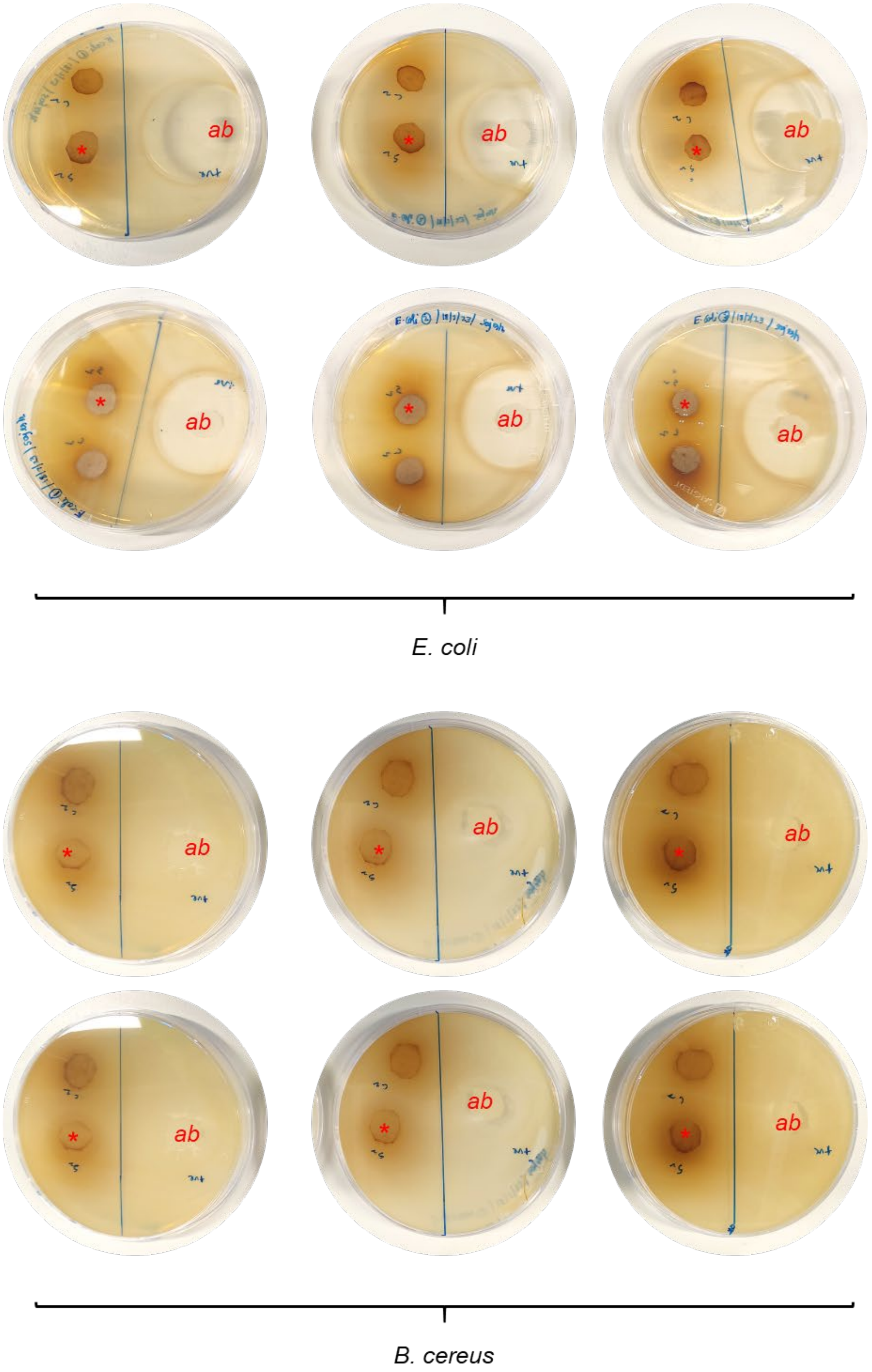

Fig. S7. Digital photographs of antibacterial test plates. Inhibition zones are lacking around the discs conditioned with lignin gel emulsions (marked with asterisks) while inhibition zones are present around the antibiotic-containing discs (marked with ab).

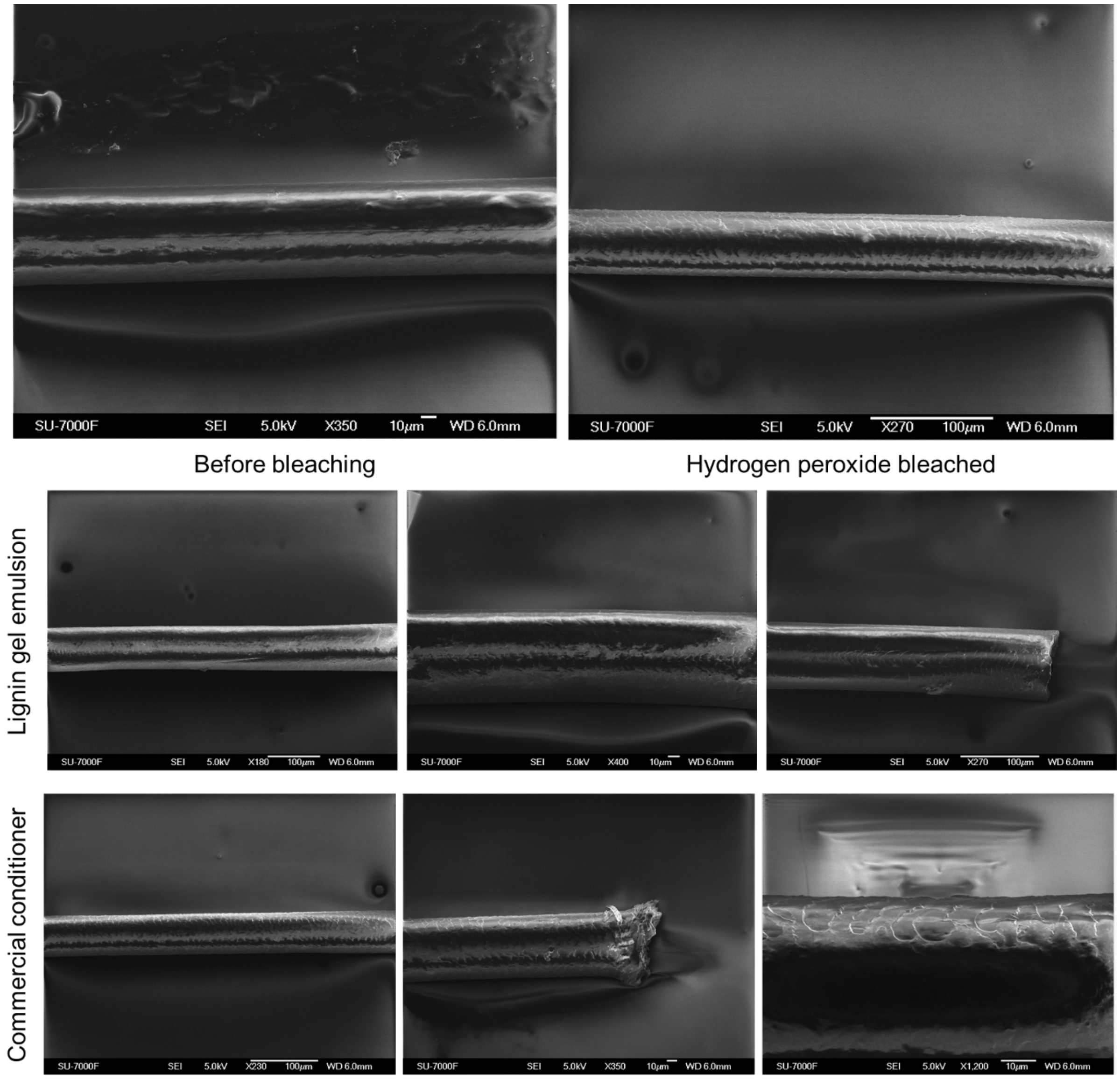

Fig. S8. SEM images of hair samples. Hair filament before and after bleaching with hydrogen peroxide, and after conditioning the H_2O_2-bleached hair with lignin gel emulsion or commercial conditioner.

Table S1. Ingredients of the commercial hair conditioner used in this study.

Ingredient	Role
Water	Solvent
Cetearyl alcohol	Emulsification and thickening (Nonionic surfactant)
Behentrimonium chloride	Conditioning agent (Cationic surfactant)
Cetyl esters	Thickening and skin conditioning
Coconut oil	Emollient – Moisturizing and hydrating agent
Sodium benzoate	Preservative (antimicrobial agent)
Phenoxyethanol	Preservative (antimicrobial agent)
Paeonia officinalis flower extract	Natural Moisturizing and hydrating agent
Ethylhexyl salicylate	UV protection (SPF)
Tridecth-6	Nonionic surfactant for emulsification
Chlorhexidine digluconate	Antimicrobial and disinfectant
Benzyl alcohol	Preservative (antimicrobial)
Benzyl salicylate	Fragrance and UV protection
Linalool	Fragrance
Amodimethicone	Conditioning agent (cationic)
Isopropyl alcohol	Quick drying
Alpha-isomethyl ionone	Fragrance
Geraniol	Antioxidant and Anti-inflammatory
Citric acid	pH adjuster
Potassium hydroxide	pH adjuster
Citronellol	Fragrance
Cetrimonium chloride	Conditioning agent (Cationic surfactant)
Potassium sorbate	Preservative (antimicrobial)
Hexyl cinnamal	Aroma
Glycerin	Moisturizing and conditioning agent
Parfum/Fragrance	Fragrance

Source: https://www.lorealparis.se/elvital/color-vive/balsam-200ml

Table S2: Water contact value of untreated and treated hair samples.

Sample name	Water contact angle		
Hair	113°	115°	117°
Bleached hair	105°	101°	101°
Bleached hair + commercial conditioner	97°	96°	96°
Bleached hair + lignin conditioner	91°	86°	86°

Video S1.

Colorimetric antioxidant activity test on lignin conditioner-treated hair.

Video S2.

Colorimetric antioxidant activity test on commercial conditioner-treated hair, and comparison to the end result of the test made using lignin conditioner-treated hair.
